# A systems biology approach for investigating significantly expressed genes among COVID-19, hepatocellular carcinoma, and chronic hepatitis B

**DOI:** 10.1186/s43042-022-00360-3

**Published:** 2022-10-20

**Authors:** Babak Sokouti

**Affiliations:** Biotechnology Research Center, Tabriz University of Medical Sciences, Tabriz, Iran

**Keywords:** COVID-19, Hepatocellular carcinoma, Chronic hepatitis B, Immune cell infiltration, Viral infection, System biology analysis, Disease interconnection

## Abstract

**Background:**

Worldwide, COVID-19’s death rate is about 2%, considering the incidence and mortality. However, the information on its complications in other organs, specifically the liver and its disorders, is limited in mild or severe cases. In this study, we aimed to computationally investigate the typical relationships between liver-related diseases [i.e., hepatocellular carcinoma (HCC), and chronic hepatitis B (CHB)] and COVID-19, considering the involved significant genes and their molecular mechanisms.

**Methods:**

We investigated two GEO microarray datasets (GSE164805 and GSE58208) to identify differentially expressed genes (DEGs) among the generated four datasets for mild/severe COVID-19, HCC, and CHB. Then, the overlapping genes among them were identified for GO and KEGG enrichment analyses, protein–protein interaction network construction, hub genes determination, and their associations with immune cell infiltration.

**Results:**

A total of 22 significant genes (i.e., ACTB, ATM, CDC42, DHX15, EPRS, GAPDH, HIF1A, HNRNPA1, HRAS, HSP90AB1, HSPA8, IL1B, JUN, POLR2B, PTPRC, RPS27A, SFRS1, SMARCA4, SRC, TNF, UBE2I, and VEGFA) were found to play essential roles among mild/severe COVID-19 associated with HCC and CHB. Moreover, the analysis of immune cell infiltration revealed that these genes are mostly positively correlated with tumor immune and inflammatory responses.

**Conclusions:**

In summary, the current study demonstrated that 22 identified DEGs might play an essential role in understanding the associations between the mild/severe COVID-19 patients with HCC and CHB. So, the HCC and CHB patients involved in different types of COVID-19 can benefit from immune-based targets for therapeutic interventions.

**Supplementary Information:**

The online version contains supplementary material available at 10.1186/s43042-022-00360-3.

## Background

SARS-CoV-2 infection is a kind of coronavirus that infects the human respiratory system in China; this type of virus first appeared in December 2019. The World Health Organization (WHO) designated its related disease as a pandemic on March 11, 2020. Over 620 million cases and nearly 6.6 million fatalities were recorded by October 14, 2022 (i.e., https://covid19.who.int/). Elderly patients with significant health problems, including diabetes, obesity, and chronic renal disease, suffer from worse disease and higher death rates [1, 2, 3]. There is a considerable variation in clinical presentations of COVID-19, which may be anything from shortness of breath to kidney problems [4, 5].

Some researchers are very concerned about the effects of COVID-19 and those connected to patients with hepatitis C or B or both diseases. Such issues often strike those with liver problems, especially those with liver fibrosis, damage, or cancer [6, 7]. And, as the number of SARS-CoV-2 and HBV or HCV coinfected individuals increases, so does the risk of creating an outbreak [8, 9]. To provide better care for the significant population of patients at risk for having HCV or HBV, understanding the liver symptoms and conditions of COVID-19 that these patients experience is essential.

Two worldwide viral infections need scrutiny: COVID-19 and HBV. The studies have indicated that hepatitis B infection does not seem to increase one’s chance of developing SARS-CoV-2. Although there are few conflicting results on the effects of chronic hepatitis B (CHB) on COVID-19 patients, we know that it may cause certain complications. COVID-19 had a greater death rate in individuals with chronic liver disease, and it needs additional investigation for confirmation [10, 11].

A recent study reveals that SARS-CoV-2 has a specific receptor on host cells, ACE2, and a strong binding capacity to the virus [2, 12, 13]. Higher expression levels in various organs seem more easily infected by SARS-CoV-2. SARS-CoV-2 is highly contagious in cancer patients, and their prognosis is bleak [14]. Additionally, patients with HCC had a decreased ACE2 expression compared to normal samples, which correlated with a poor prognosis [14]. Nevertheless, many questions remain about the role that the SARS-CoV-2 proteins play in regulating human mRNA expression in cancer.

The fact that earlier research has shown that COVID-19 has a relationship to liver function in people with chronic hepatitis B has not deterred researchers from investigating its role in people with other liver disorders (e.g., CHB). Moreover, researchers published the findings on people with persistent HBV infections who had COVID‐19. On the other hand, various biomarker signatures may serve as diagnostic information to enhance treatment plans for HCC and COVID-19 in specific personalized medicine.

## Methods

### Microarray datasets

Data from the NCBI-Gene Expression Omnibus (GEO) database, GSE164805 and GSE58208, were used in this study with a total sample size of 42. The 15 samples from GSE164805 come from five severe COVID-19 patients, five mild COVID-19 patients, and five healthy samples. There were ten individuals with HCC, twelve patients with CHB in GSE58208, and five control samples (i.e., GSE58208 has both CHB and HCC samples separately without in-common patients involved). The control groups, who were a reference in the datasets, had no inflammation, viral genome, or HCC/CHB histories. The GSE164805 samples were run on the Agilent-085982 Arraystar human lncRNA V5 microarray (GPL26963) to produce the expression profiling arrays, whereas the GSE58208 samples were run on the Affymetrix Human Genome U133 Plus 2.0 Array (GPL570).

### Identification of DEGs

By comparing patients infected with mild and severe SARS-CoV-2, CHB, and HCC and their corresponding control samples, differentially expressed genes (DEGs) were identified using the GEO2R tool as well as “umap,” “GEOquery,” and “limma” packages [15]. Next, we selected the statistically significant DEGs (|log_2_(FoldChange)|≥ 0.5) with a *p* value of less than 0.05 and Benjamini & Hochberg (False discovery rate) criterion for *p* value adjustment. Last, we used the Multiple List Comparator free publicly available tool in the Web site link (i.e., http://www.molbiotools.com/) to create Venn diagrams that show the overlapped DEGs obtained for the diseases mentioned above.

### GO and KEGG enrichment analyses

Gene Ontology (GO) and KEGG (Kyoto Encyclopedia of Genes and Genomes) pathway enrichment analyses were performed for the obtained significant DEGs. A major Knowledgebase upgrade, DAVID (Database for Annotation, Visualization, and Integrated Discovery) v6.8, comprises a complete collection of tools to interpret the meaning of genes with powerful annotation capabilities useful for scientists [16]. The GO analysis includes a biological process (BP), molecular functions (MF), and cellular components (CC). Additionally, the KEGG pathway analysis was carried out. The threshold of *p* value < 0.05 was used for both GO enrichment and KEGG pathway analyses.

### Protein–protein interaction network construction and hub genes identification

The protein–protein interaction (PPI) network was generated, analyzed, and visualized using the Cytoscape 3.7.1 through STRING program (with default parameters) and included all the known interactions among the cell’s proteins [17, 18]. We employed the Cytoscape cytoHubba plugin to investigate the associations between the DEGs, including analyzing, clustering, and identifying hub genes. Using the plugin, the “Degree” criterion was selected for hub genes identification. Finally, the 10 top essential genes were picked from the PPI network analysis that can reveal the critical hub genes.

### Assessment of immune cell infiltration

We utilized the TIMER (Tumour Immune Estimation Resource) database available through the R-based Web site link as a shiny app to estimate the correlations between the mRNA expression data for identified DEGs and tumor purity and the quantity of immunological infiltrates, as well as B cells, CD4^+^ T cells, CD8^+^ T cells, neutrophils, macrophages, and dendritic cells. There were almost 10,000 samples from the TCGA representing 32 different kinds of cancer in this database [19, 20, 21]. All data were analyzed using the Spearman correlation technique where the categorization correlation criteria are as follows: low 0.00–0.19; moderate 0.20.03–0.39; high 0.40.04–0.59; very high 0.60.0–1.0.

## Results

### DEGs identification

In total, 19,336, 21,774, 4294, and 4663 DEGs were extracted from the GSE164805, GSE164805, GSE58208, and GSE58208 microarray datasets, respectively. And the results indicated that 2328, 2389, 2212, and 2487 DEGs were in common among different datasets: severe COVID-19 and CHB, mild COVID-19 and HCC, mild COVID-19 and CHB, and severe COVID-19 and HCC, respectively (Additional file 1: Fig S1).

### Enrichment analyses of GO and KEGG

**Fig. 1:**
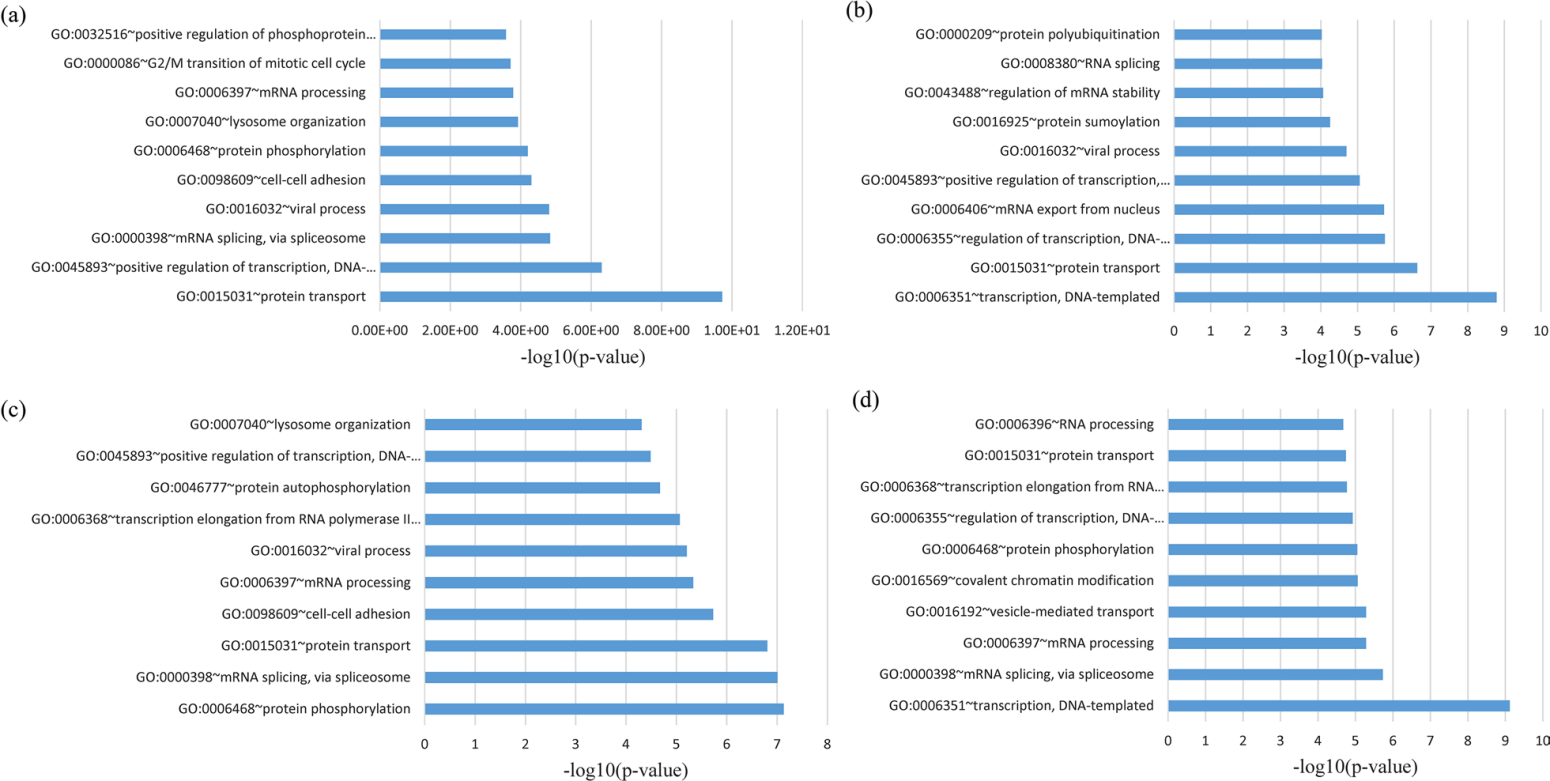
GO biological process enrichment analysis of overlapping DEGs: **a** mild COVID-19 versus CHB, **b** mild COVID-19 versus HCC, **c** severe COVID-19 versus CHB, **d** severe COVID-19 versus HCC datasets

The DAVID Web site was used to run the GO and KEGG pathway enrichment investigation, from which the illustrated outcomes were then shown in terms of -log_10_ (*p* value). The DEGs were grouped according to their GO analysis into MF, BP, and CC. According to the BP analysis, the shared genes between the four datasets were primarily associated with protein transport and protein autophosphorylation. In contrast, the shared genes between mild COVID-19 and CHB and HCC were associated with protein transport, positive regulation of transcription DNA-templated, and transcription DNA-templated. Moreover, the shared genes between severe COVID-19 and CHB and HCC were related to protein phosphorylation, mRNA splicing via spliceosome, and transcription DNA template (Fig. 1). For the CC analysis, the shared DEGs among four datasets were mainly enriched in neoplasm, cytoplasm, and membrane, while the shared DEGs between mild COVID-19 and CHB as well as HCC were located in membrane and neoplasm.

**Fig. 2:**
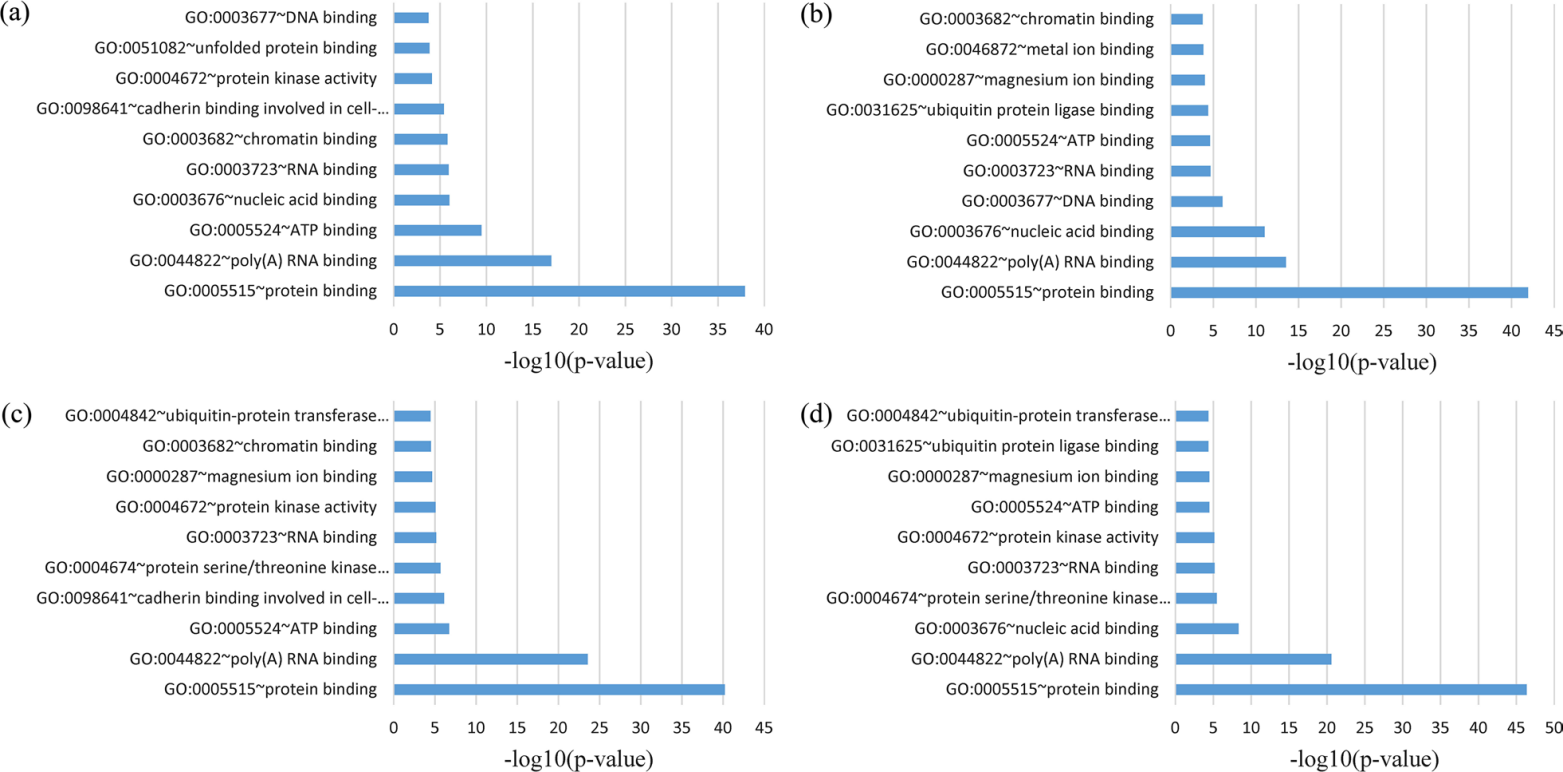
GO cellular component enrichment analysis of overlapping DEGs: **a** mild COVID-19 versus CHB, **b** mild COVID-19 versus HCC, **c** severe COVID-19 versus CHB, **d** severe COVID-19 versus HCC datasets

**Fig. 3:**
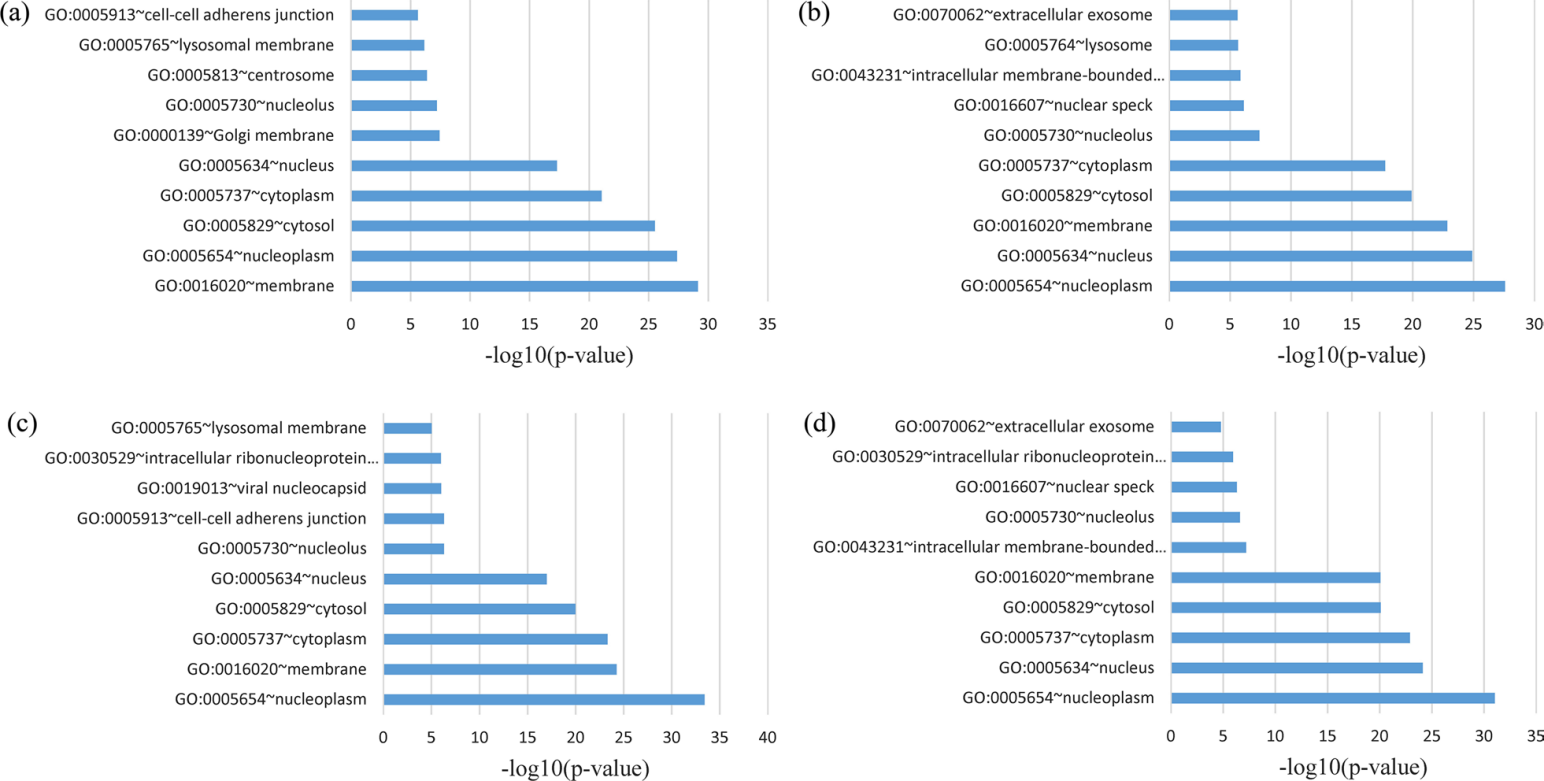
GO molecular function enrichment analysis of overlapping DEGs: **a** mild COVID-19 versus CHB, **b** mild COVID-19 versus HCC, **c** severe COVID-19 versus CHB, **d** severe COVID-19 versus HCC datasets

**Fig. 4:**
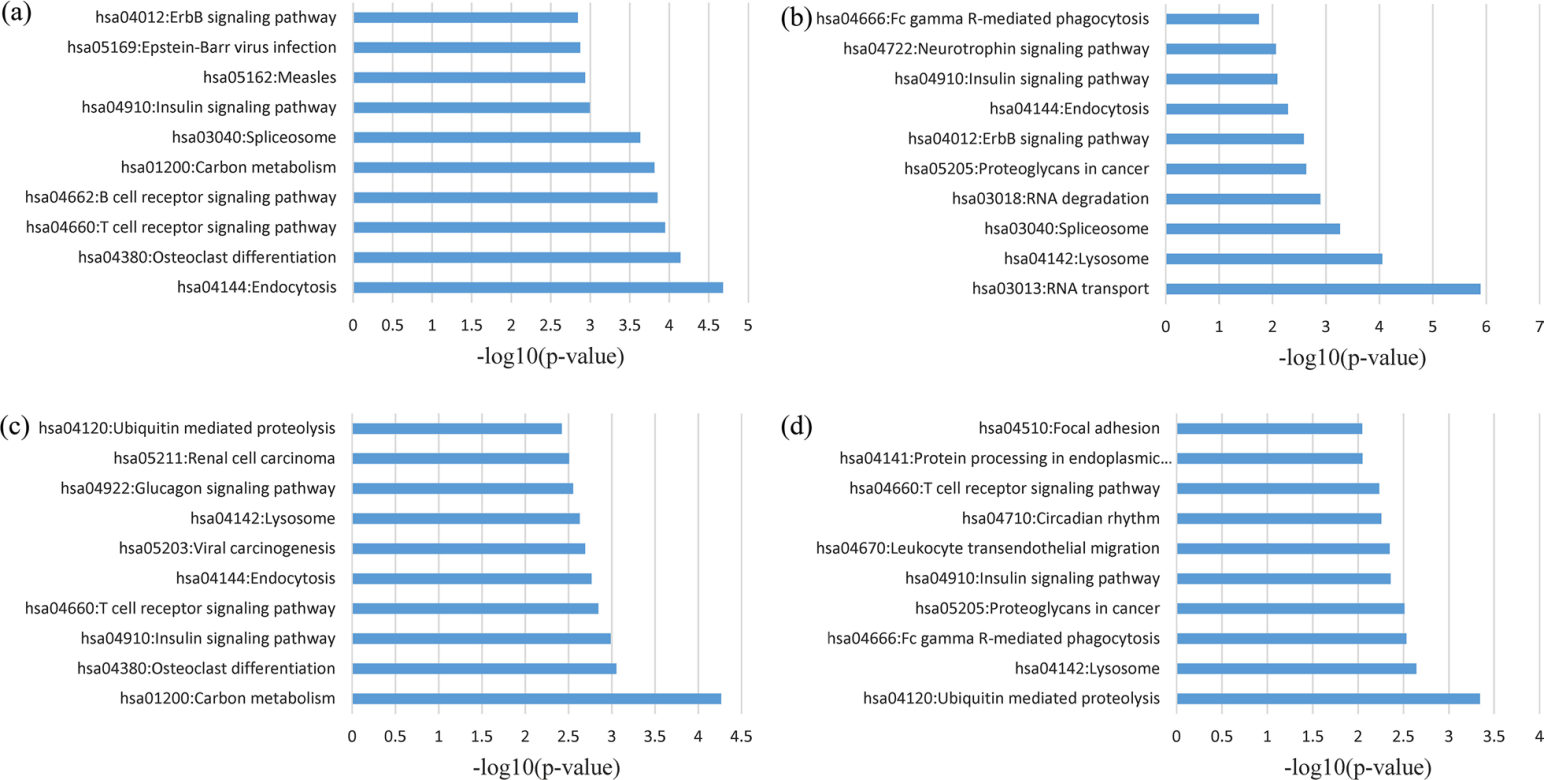
KEGG signaling pathways enrichment analysis of overlapping DEGs: **a** mild COVID-19 versus CHB, **b** mild COVID-19 versus HCC, **c** severe COVID-19 versus CHB, **d** severe COVID-19 versus HCC datasets

Additionally, the shared genes between severe COVID-19 and CHB as well as HCC were positioned at the neoplasm, nucleus, and membrane (Fig. 2). In the MF, the shared gene among four datasets was primarily enriched in protein binding and poly(A) RNA binding. At the same time, common genes between mild/severe COVID-19 and CHB as well as HCC were also increased in protein binding and poly(A) RNA binding (Fig. 3). Genes frequent in the KEGG signaling pathway analysis were also shown to be enriched in the insulin signaling pathway, endocytosis, RNA transport, carbon metabolism, and ubiquitin-mediated proteolysis (Fig. 4).

### Hub genes identification

**Fig. 5:**
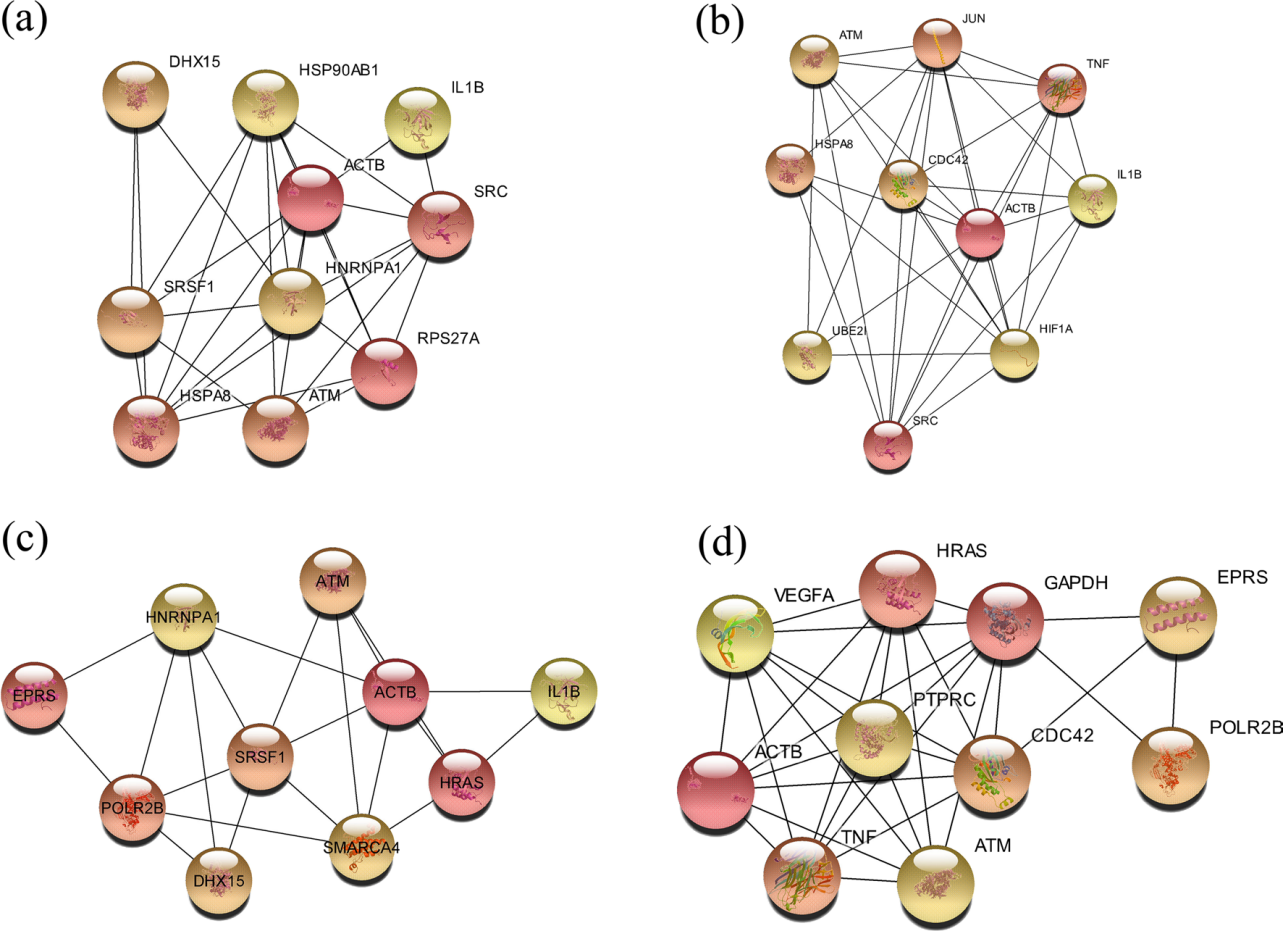
Correlation between the identified DEGs and immune cell infiltration (TIMER); *p* value < 0.05 represented statistically significant

The PPI network of the determined DEGs for severe COVID-19 versus HCC (2389 nodes and 25,819 edges), severe COVID-19 versus CHB (2264 nodes and 24,656 edges), mild COVID-19 versus HCC (2292 nodes and 22,386 edges), and mild COVID-19 versus CHB (2139 nodes and 21,939 edges) was constructed. The top 10 genes were identified using the 11 techniques provided in cytoHubba and ranked using the degree method. The identified hub gene sets for severe COVID-19 versus HCC, severe COVID-19 versus CHB, mild COVID-19 versus HCC, and mild COVID-19 versus CHB are shown in Fig. 5.

### Significant DEGs associated with immune cell infiltration

The statistically significant existence of either negative or positive correlations between identified unique DEGs from the previous section and immune cell infiltration was comprehensively explored based on the TIMER database. The results revealed that there was closely a positive correlation between the expression of all DEGs and the infiltration of B cells, CD8^+^ T cells, CD4^+^ T cells, macrophages, neutrophils, and dendritic cells (*p* < 0.05). The JUN, JUN, and GAPDH expressions were not significantly correlated for infiltration of B cells, CD8^+^ T cells, and CD4^+^ T cells, respectively. Moreover, no significant correlation was identified between the expression of all DEGs and the purity. However, some of the target DEGs identified negative (i.e., ACTB, IL1B, PTPRC, SRC, TNF) and positive (HNRNPA1, VEGFA) correlations. More details are depicted in Additional file 2: Fig S2.

## Discussion

About 391 million persons globally are at risk of developing decompensated liver disease and hepatocellular cancer due to CHB [22]. Preexisting liver disease comorbidity may impact the results of COVID-19 in patients with chronic viral hepatitis [20]. Oncologists worldwide were affected by the COVID-19 epidemic. In this pandemic, CHB may be significant comorbidity of preexisting liver illnesses. A study suggests that HCC sufferers are more likely to develop severe COVID-19 because they have both cancer and chronic liver disease at the same time; however, there is no available clinical evidence [23]. Strengthening one's immune system via regular physical activity is linked to a better response to viral infectious illnesses like COVID-19 [24].

Identifying undesirably expressed candidate genes, which may provide insight into how a process is regulated in response to a stimulus, may be of considerable assistance in determining the appropriate therapy targets and preventive measures to take. Accordingly, in this work, bioinformatics methods were employed to examine two microarray datasets (GSE164805 and GSE58208) to find probable drivers. Using the utilized criteria, we were able to narrow down the list of candidate hub genes to 22 (ACTB, ATM, CDC42, DHX15, EPRS, GAPDH, HIF1A, HNRNPA1, HRAS, HSP90AB1, HSPA8, IL1B, JUN, POLR2B, PTPRC, RPS27A, SFRS1, SMARCA4, SRC, TNF, UBE2I, VEGFA) that will be discussed in detail as follows.

For comparisons across species and tissues, the researchers used the ACTB gene expression data from each of their RNA-seq experiments to normalize the expression of ACE2. Most cell types produced the housekeeping gene ACTB, which was an internal control for gene expression measurements. Additionally, all species investigated have the ACTB gene [25].

TLR7-ATM deficiency may cause two distinct immunodeficiency phenotypes. The potential that the ATM deficit influenced the progression of COVID-19 in the TLR7-deficient case cannot be ruled out. Furthermore, the death of B cells due to ATM deficiency reduced germinal center frequency and size after vaccination. Patients with COVID-19 pneumonia who have X-linked TLR7 and ATM pathogenic mutations, along with their clinical and molecular characterizations, have also been studied elsewhere [26].

Various signaling pathways could target CDC42, which was downregulated in HCC515 cells with COL-3 treatment [27]. CDC42 could also be affected by the Coronaviridae family neurotropic virus (i.e., porcine hemagglutinating encephalomyelitis virus) [28]. Additionally, CDC42 is one of the many genes downregulated in lung cells when COL-3 functions as a protective mechanism. So, such genes (CDC42) are required for a chemokine, TNF, and MAPK signaling pathways [27].

It is crucial to note that SARS-CoV-2 is an RNA virus that is the source of the current global coronavirus epidemic [29, 30]. SARS-CoV-2 may readily infect human intestinal epithelial cells in individuals with COVID-19 who have gastrointestinal symptoms and fecal RNA excretion of the virus [31, 32]. RNA helicase DHX15 may be targeted to regulate SARS-CoV-2 and the inflammation of the gastrointestinal tract produced by SARS-CoV-2, based on the findings on its involvement in regulating enteric RNA virus-induced intestinal inflammation [33].

The mRNA vaccine vesicles must enter cells and hijack the host cell machinery to create a viral protein antigen, which will then be processed and presented by MHC molecules to stimulate B and T cell responses. All are necessary to translate mRNA, the ribosome, translation initiation factors, aminoacyl-tRNA synthetases, and elongation factors. EPRS seems to control pro-fibrotic protein production [34].

The ACE2 mRNA transcript level was determined to be comparable to GAPDH and beta-actin. Overall, the data show that the mRNA encoding for the ACE2 gene is expressed similarly across patients. Patients with COVID-19 will need to be investigated in the future to learn more about how SARS-CoV-2 infection affects the thyroid [35].

ACE2 expression was lowered by HIF1A knockdown under hypoxic circumstances, alone or combined [36]. Patients with severe COVID-19 may also see an increase in the degradation of FOXP3 proteins because the hypoxic lung environment activates HIF1A, which is involved in aerobic glycolysis [37]. Furthermore, it was shown that overexpression of HIF1A in human embryonic kidney cells strongly decreases the production of ACE2 [38].

HNRNPA1 was downregulated in Calu-3 cells, indicating a human cell's reaction to inhibit viral replication in contrast to the prior instances of upregulated genes. Human RNA-binding proteins such as HNRNPA1, which was recently identified as the “hub protein” with the most significant functional ties to the human SARS-CoV-2 genome, were shown to have conserved connections to the SARS-CoV-2 genome in this research [39, 40].

In addition to playing a significant part in cancer development, the signaling of growth factor receptors (GFR) is also essential for the progression of some viral infections [41–43]. Recent investigations have shown that infection with SARS-CoV-2 significantly alters the expression of numerous genes involved in cellular signaling pathways essential in cancer development. One of these is the RAS-RAF/MEK/ERK signaling pathway [44]. Those with COVID-19 and more severe symptoms have been shown to have more significant H-Ras gene overexpression in their peripheral blood mononuclear cells (PBMC).

The SARS-CoV-2 infection triggers such overexpression in immune cells, a molecular process. This finding can highlight the importance of the Ras family of genes in the host immune response, not just to COVID-19 but to other viral infectious diseases as well [45].

Viruses may activate Hsps, particularly Hsp70 and Hsp90, which increase viral replication. For example, the Hsp90 inhibitor geldanamycin inhibits coronavirus replication in cell culture systems [46]. HSP90AB1 overexpression also suggests that increased inflammation triggered extrapulmonary tissue remodeling. The estrogen signaling pathway connected with the HSP90AB1 target was identified as the primary COVID-19 signaling mechanism [47].

The HSPA8 protein involved in antigen processing and presentation was changed in six COVID-19 individuals [48]. HSPA8 was one of the most downregulated COVID-19 genes [49]. The results demonstrated that HSPA8 was connected to other genes involved with heat shock response and ER stress, suggesting that cellular stress response is vital in SARS-CoV2-related coronavirus pathogenesis [46]. Also, in SARS-CoV-2-infected NHBE cells, many interleukin genes were elevated, including IL1B [50]. SARS-CoV-2-infected NHBE cells exhibited a significant increase in lung fibrosis-related genes (e.g., IL1B). For COVID-19 individuals suffering from an inflammatory storm, an integrated analysis suggested that new target genes such as IL-1 may be beneficial [51].

Cardiac myocyte hypertrophy is linked to an increase in c-Jun expression level, according to research. As part of the activator protein-1 complex, c-Jun regulates cell death and survival [52]. Viral infections are connected with inflammation, and JUN is crucial in this process [40]. In individuals with COVID-19, CD4^+^ T cells expressed significant amounts of inflammatory genes, including JUN [53].

A topological study of the gain component was carried out, and the POLR2B gene, which was shown to have a higher network degree, was discovered [54]. The POLR2B gene was shown to be ubiquitinated, and its protein levels dropped in other studies [55]

As a critical antigen, T cell activation in atherosclerosis relies on PTPRC, previously known as CD45. Vaccination may activate nave B cells, as shown by the upregulation of many genes (such as PTPRC) implicated in GO pathways for B cell activation. Severely affected COVID-19 patients have lower levels of the PTPRC gene, indicating that activation of nave B cells may be hampered in these people [56, 57].

The housekeeping Rps27a gene encodes a 40S subunit 20 of the ribosomes. There are two protein-coding transcripts for the mouse Rps27a gene (named S27a-44 and S27a-45). It was discovered that the levels of two of the four ribosomal fusion proteins encoded by human ubiquitins, RPS27A and UBA52, were elevated in patients with Alzheimer's disease and COVID-19 with Alzheimer's disease, respectively [28, 58].

A genome-wide CRISPR investigation has identified SMARCA4 as a SARS-CoV-2-specific gene. SMARCA4 and SMAD3 may be involved in SARS-CoV-2 entrance, and virus-induced cell death is possible. The proviral activity of SMARCA4, a protein expressed by the SMAD3 gene, has been described by Wei's group [59].

However, ABL kinases are combined with SRC family kinases to enhance vaccinia virus actin-based motility. Patients infected with SARS-CoV-2 may benefit from SRC's anti-inflammatory and antifibrotic action and cytokine inhibition. RPS3 was found to be an essential player in viral replication, while SRC non-receptor protein kinase was found to be a hub gene in the inflammatory response. It has been observed that proteins like SRC, which control the cytokine secretion network, were crucial in the ACE2-related inflammatory responses to the cytokine secretion-associated proteins [60, 61].

In the COVID-19 cohort with Alzheimer's disease, the serine- and arginine-rich splicing factor 1 is encoded by the downregulation of SRSF1. SRSF1 activity becomes uncontrolled due to neurodegeneration-related hypoxia, resulting in increased splicing, which is then detected in Alzheimer's patients [28].

High levels of TNF-α are caused, according to Tsukamoto et al. [62], by genetic changes at the TNF-α locus. Increased TNF-α production is linked to two biallelic polymorphisms in TNF-α, one associated with severe infectious diseases [63]. Consequently, anti-TNF medicine is being considered a possible treatment for COVID-19 [57]. Inflammatory cytokines like TNF and IFNg have been linked to low vitamin D levels. In contrast, vitamin D has been shown to suppress these cytokines and boost anti-inflammatory cytokines generated by macrophages [51].

UBE2I is a ubiquitin-conjugating enzyme involved in protein breakdown, although it may also promote transcription [64].

Due to the removal of ACE2's antagonistic effect on VEGFA production, SARS-CoV-2 elevates levels of VEGFA in the body. Heart problems are also significantly linked to the rise in VEGFA expression, which causes cardiac hypertrophy to proceed. For example, ACE2 suppresses cell invasion and migration in NSCLC cells by suppressing VEGFA/VEGFR2/ERK pathway in breast cancer. Antagonizing VEGFA has also been caused by SARS-CoV-2 downregulation of ACE2, an essential factor. VEGFA is a crucial gene in COVID-19 cancer high-risk patients, according to a comprehensive gene–disease association analysis [52, 65, 66].

When the livers of COVID-19 patients who had passed away were examined histologically, it was discovered that there were extensive vascular abnormalities, steatosis, and mitochondrial abnormalities. These conditions were assumed to be brought on by SARS-CoV-2 [10]. According to the findings of several studies, the prognosis for patients with COVID-19 infections was poorer when the patients also had an associated chronic liver illness, which reduced the patients' liver function [66–68]. Extremely high levels of angiotensin-converting enzyme 2 (ACE2) have been seen in many different kinds of cancer cells, including lung adenocarcinoma (LUAD) and lung squamous carcinoma (LUSC), suggesting a link between COVID-19 and the progression of cancer and the mortality rate [70]. It is possible to indicate that the likelihood of direct liver damage of the HCC patient by SARS-CoV-2 would be unlikely owing to the low expression of ACE2. Still, HCC patients with obesity are more likely to be affected by COVID-19-linked severe pathogenesis and poor prognosis [71].

Twenty percent of cirrhotic patients with COVID-19 had acute-on-chronic liver failure or acute decompensation, according to a global study that included cohorts from 13 Asian countries [69]. This is a significant result because it indicates that persons with cirrhosis are at increased risk of experiencing severe liver damage from the SARS-CoV-2 virus. Patients infected with COVID-19 who already have liver disease have a more significant risk of severe complications and mortality than healthy patients [72]. According to the findings of a research that included 15 patients with chronic hepatitis B and COVID-19, those patients had a more severe illness and a higher death rate when compared to patients who did not have HBV infection, which suggests that HBV coinfection may increase the development of liver damage, which is linked with unfavorable outcomes [73, 74].

Moreover, many researchers have investigated potential relationships between COVID-19 and chronic liver disease. However, the roles of these 22 genes are important while being related to the clinical manifestations.

To understand the pathophysiological and clinical features of SARS-CoV-2 infection, one may need to have an in-depth comprehension on cytokine storm because of inappropriate recognition of the pathogen with an improper response of the immune system involving different genes and signaling pathways [75]. Furthermore, the immune response at the liver may result in immunodeficiency in patients with liver disease by distorting the liver architecture and hence disarrange the cellular organization and functions resulting in a hepatic inability to synthesize proteins. By taking into account that inflammatory cytokine signaling in the immune system and IL signaling (VEGFA, TNF, SMARCA4, IL1B, JUN, HSP90AB1, HIF1A) and B cell/T cell receptor signaling pathway (JUN, PTPRC) play important roles through the cytokine storm. As, patients with COVID-19 often have multi-organ failure, which may include liver damage due to the hypoxia and cytokine storm that accompanies SARS-CoV-2 infection [75, 76]. As also seen in COVID-19, cytokine storm has the potential to be engaged in disseminated intravascular coagulation (DIC) and thrombocytopenia, both of which have the potential to aggravate the effects of DIC [77].

On the other hand, multiple signaling pathways may be involved in both diseases such as MAPK (CDC42, HRAS, HSP90AB1, HSPA8, RPS27A, SRC), PI3K-Akt-mTOR (HRAS, HSP90AB1), hypoxia (ACTB, JUN, VEGFA, HIF1A, GAPDH), JAK/STAT (PTPRC, SRC, UBE2I), and NF-kB signaling pathways (ATM, HRAS, IL1B, JUN, PTPRC, SFRS1, TNF) that are the key factors in these patients with hypercoagulation, infection, and inflammation [11, 78].

Last but not least, apoptosis pathway (ACTB, ATM, HSPA8, SFRS1, TNF, UBE2I), DNA damage and metabolism of RNA (CDC42, JUN, POLR2B, SMARCA4, DHX15, EPRS, HNRNPA1), and cell cycle process (ATM, POLR2B, UBE2I, VEGFA) can play roles in hepatocellular necrosis and cellular infiltration in the liver organ of patients with COVID-19, resulting in liver apoptosis [79].

Gene expression analysis has contributed to personalized medicine advancements in cancer and transplantation. This has given us a reason to apply similar methods to other diseases, such as those related to prototypical inflammatory autoimmune diseases with unknown causes. Increased understanding of disease biology, better patient care, and more tailored therapy approaches are only some of the potential benefits of gene expression profiling's clinical and translational use. Once a novel biomarker or combination of biomarkers has been discovered in fundamental research, it may take some time until they are used in clinical practice.

This is due to the time and effort needed to validate biomarkers, establish their practical efficacy, develop a manufacturing method, and get regulatory approval. When taken together, these results highlight gene expression's worth as a novel tool with the potential to provide unique data to each person [80, 81].

## Conclusions

A total of 22 hub genes were identified among HCC, CHB, and mild/severe COVID-19 after evaluating two GEO datasets utilizing existing methods for GO and KEGG enrichment approaches, construction of a PPI network for hub gene identification, and immune cell infiltration. These genes may have the highest potential role in signaling pathways, biological processes, cellular components, molecular functions, and immunological and inflammatory processes. Considering the outcomes obtained from the above-mentioned approach among HCC, CHB, and mild/severe COVID-19, it is vital to study the disease interconnections in-depth soon. And more immune-based therapeutic targets can be developed to cover the potential gaps specifically in terms of their molecular mechanisms among these illnesses.

## Supplementary Information

**Additional file 1**. **Fig 1**: Venn diagrams depicting the common DEGs between mild COVID-19 vs. CHB, mild COVID-19 vs. HCC, severe COVID-19 vs. CHB, severe COVID-19 vs. HCC datasets

**Additional file 2**. **Fig 2**: Correlation between the identified DEGs and immune cell infiltration (TIMER); p value<0.05 represented statistically significant

## Data Availability

Not applicable.

## References

[1] Földi M, Farkas N, Kiss S, Zádori N, Váncsa S, Szakó L et al (2020) Obesity is a risk factor for developing critical condition in COVID-19 patients: a systematic review and meta-analysis. Obes Rev Off J Int Assoc Study Obes 21(10):e13095. 10.1111/obr.13095 10.1111/obr.13095 PMC7404429 32686331

[2] Guo W, Li M, Dong Y, Zhou H, Zhang Z, Tian C et al (2020) Diabetes is a risk factor for the progression and prognosis of COVID-19. Diabetes/Metab Res Rev. 10.1002/dmrr.3319 10.1002/dmrr.3319 PMC7228407 32233013

[3] Wang L, He W, Yu X, Hu D, Bao M, Liu H et al (2020) Coronavirus disease 2019 in elderly patients: characteristics and prognostic factors based on 4-week follow-up. J Infect 80(6):639–645. 10.1016/j.jinf.2020.03.019 32240670 10.1016/j.jinf.2020.03.019 PMC7118526

[4] Goyal P, Choi JJ, Pinheiro LC, Schenck EJ, Chen R, Jabri A et al (2020) Clinical characteristics of Covid-19 in New York City. N Engl J Med 382(24):2372–2374. 10.1056/NEJMc2010419 32302078 10.1056/NEJMc2010419 PMC7182018

[5] Khalili M, Karamouzian M, Nasiri N, Javadi S, Mirzazadeh A, Sharifi H (2020) Epidemiological characteristics of COVID-19: a systematic review and meta-analysis. Epidemiol Infect 148:e130. 10.1017/s0950268820001430 32594937 10.1017/S0950268820001430 PMC7343974

[6] Hernandez Mdel P, Martin P, Simkins J (2015) Infectious complications after liver transplantation. Gastroenterol Hepatol 11(11):741–753 PMC4849501 27134589

[7] Kunutsor SK, Laukkanen JA (2020) Hepatic manifestations and complications of COVID-19: a systematic review and meta-analysis. J Infect 81(3):e72–e74. 10.1016/j.jinf.2020.06.043 32579984 10.1016/j.jinf.2020.06.043 PMC7306105

[8] Blach S, Zeuzem S, Manns M, Altraif I, Duberg AS, Muljono DH, Waked I, Alavian SM, Lee MH, Negro F, Abaalkhail F (2017) Global prevalence and genotype distribution of hepatitis C virus infection in 2015: a modelling study. Lancet Gastroenterol Hepatol. 2(3):161–176. 10.1016/s2468-1253(16)30181-9 28404132 10.1016/S2468-1253(16)30181-9

[9] Razavi-Shearer D, Gamkrelidze I, Nguyen MH, Chen DS, Van Damme P, Abbas Z, Abdulla M, Abou Rached A, Adda D, Aho I, Akarca U (2018) Global prevalence, treatment, and prevention of hepatitis B virus infection in 2016: a modelling study. Lancet Gastroenterol Hepatol. 3(6):383–403. 10.1016/s2468-1253(18)30056-6 29599078 10.1016/S2468-1253(18)30056-6

[10] Marjot T, Moon AM, Cook JA, Abd-Elsalam S, Aloman C, Armstrong MJ et al (2021) Outcomes following SARS-CoV-2 infection in patients with chronic liver disease: an international registry study. J Hepatol 74(3):567–577. 10.1016/j.jhep.2020.09.024 33035628 10.1016/j.jhep.2020.09.024 PMC7536538

[11] Martinez MA, Franco S (2021) Impact of COVID-19 in liver disease progression. Hepatol Commun 5(7):1138–1150. 10.1002/hep4.1745 34533001 10.1002/hep4.1745 PMC8239862

[12] Lu R, Zhao X, Li J, Niu P, Yang B, Wu H et al (2020) Genomic characterisation and epidemiology of 2019 novel coronavirus: implications for virus origins and receptor binding. Lancet (London, England) 395(10224):565–574. 10.1016/s0140-6736(20)30251-8 32007145 10.1016/S0140-6736(20)30251-8 PMC7159086

[13] Zhou P, Yang X-L, Wang X-G, Hu B, Zhang L, Zhang W et al (2020) A pneumonia outbreak associated with a new coronavirus of probable bat origin. Nature 579(7798):270–273. 10.1038/s41586-020-2012-7 32015507 10.1038/s41586-020-2012-7 PMC7095418

[14] Dai YJ, Hu F, Li H, Huang HY, Wang DW, Liang Y (2020) A profiling analysis on the receptor ACE2 expression reveals the potential risk of different type of cancers vulnerable to SARS-CoV-2 infection. Ann Transl Med 8(7):481. 10.21037/atm.2020.03.61 32395525 10.21037/atm.2020.03.61 PMC7210193

[15] Barrett T, Wilhite SE, Ledoux P, Evangelista C, Kim IF, Tomashevsky M et al (2013) NCBI GEO: archive for functional genomics data sets—update. Nucleic Acids Res 41(D1):D991–D995. 10.1093/nar/gks1193 23193258 10.1093/nar/gks1193 PMC3531084

[16] da Huang W, Sherman BT, Lempicki RA (2009) Systematic and integrative analysis of large gene lists using DAVID bioinformatics resources. Nat Protoc 4(1):44–57. 10.1038/nprot.2008.211 19131956 10.1038/nprot.2008.211

[17] Doncheva NT, Morris JH, Gorodkin J, Jensen LJ (2019) Cytoscape StringApp: network analysis and visualization of proteomics data. J Proteome Res 18(2):623–632. 10.1021/acs.jproteome.8b00702 30450911 10.1021/acs.jproteome.8b00702 PMC6800166

[18] Shannon P, Markiel A, Ozier O, Baliga NS, Wang JT, Ramage D et al (2003) Cytoscape: a software environment for integrated models of biomolecular interaction networks. Genome Res 13(11):2498–2504 14597658 10.1101/gr.1239303 PMC403769

[19] Li T, Fan J, Wang B, Traugh N, Chen Q, Liu JS et al (2017) TIMER: a web server for comprehensive analysis of tumor-infiltrating immune cells. Can Res 77(21):e108–e110. 10.1158/0008-5472.can-17-0307 10.1158/0008-5472.CAN-17-0307 PMC6042652 29092952

[20] Liu J, Wang T, Cai Q, Sun L, Huang D, Zhou G et al (2020) Longitudinal changes of liver function and hepatitis B reactivation in COVID-19 patients with pre-existing chronic hepatitis B virus infection. Hepatol Res Off J Jpn Soc Hepatol 50(11):1211–1221. 10.1111/hepr.13553 10.1111/hepr.13553 PMC7436737 32761993

[21] Li B, Severson E, Pignon JC, Zhao H, Li T, Novak J et al (2016) Comprehensive analyses of tumor immunity: implications for cancer immunotherapy. Genome Biol 17(1):174. 10.1186/s13059-016-1028-7 27549193 10.1186/s13059-016-1028-7 PMC4993001

[22] Wang J, Lu Z, Jin M, Wang Y, Tian K, Xiao J et al (2021) Clinical characteristics and risk factors of COVID-19 patients with chronic hepatitis B: a multi-center retrospective cohort study. Front Med. 10.1007/s11684-021-0854-5 34387851 10.1007/s11684-021-0854-5 PMC8362646

[23] Chagas AL, Fonseca LGD, Coelho FF, Saud L, Abdala E, Andraus W et al (2020) Management of hepatocellular carcinoma during the COVID-19 pandemic-São Paulo Clínicas liver cancer group multidisciplinary consensus statement. Clinics (Sao Paulo, Brazil) 75:e2192. 10.6061/clinics/2020/e2192 33146360 10.6061/clinics/2020/e2192 PMC7561060

[24] Bello B, Useh U (2021) COVID-19: Are non-communicable diseases risk factors for its severity? Am J Health Promot AJHP 35(5):720–729. 10.1177/0890117121990518 33576237 10.1177/0890117121990518

[25] Sun K, Gu L, Ma L, Duan Y (2021) Atlas of ACE2 gene expression reveals novel insights into transmission of SARS-CoV-2. Heliyon. 7(1):e05850-e. 10.1016/j.heliyon.2020.e05850 33392409 10.1016/j.heliyon.2020.e05850 PMC7762714

[26] Hassan A, Ahmad V, Takaki A, Nils L, Bertrand B, Samaneh D et al (2021) X-Linked TLR7 deficiency underlies critical COVID-19 pneumonia in a male patient with ataxia-telangiectasia. J Clin Immunol. 10.1007/s10875-021-01151-y 34686943 10.1007/s10875-021-01151-y PMC8536475

[27] Bing H, Lana G (2020) Prediction of repurposed drugs for treating lung injury in COVID-19. F1000Research. 10.12688/f1000research.23996.1 10.12688/f1000research.23996.1 PMC7468567 32934806

[28] Luigi C, Agnese G, Emanuela M (2021) SARS-CoV-2 exacerbates beta-amyloid neurotoxicity, inflammation and oxidative stress in alzheimer’s disease patients. Int J Mol Sci. 10.3390/ijms222413603 34948400 10.3390/ijms222413603 PMC8705864

[29] Guan WJ, Ni ZY, Hu Y, Liang WH, Ou CQ, He JX et al (2020) China medical treatment expert group for Covid19. N Engl J Med 382:1708–1720 32109013 10.1056/NEJMoa2002032 PMC7092819

[30] Zhu N, Zhang D, Wang W, Li X, Yang B, Song J et al (2020) China novel coronavirus investigating and research team. N Engl J Med 382:727–33 31978945 10.1056/NEJMoa2001017 PMC7092803

[31] Lamers MM, Beumer J, van der Vaart J, Knoops K, Puschhof J, Breugem TI et al (2020) SARS-CoV-2 productively infects human gut enterocytes. Science 369:50–4 32358202 10.1126/science.abc1669 PMC7199907

[32] Zang R, Gomez Castro MF, McCune BT, Zeng Q, Rothlauf PW, Sonnek NM et al (2020) TMPRSS2 and TMPRSS4 promote SARS-CoV-2 infection of human small intestinal enterocytes. Sci Immunol 5:3582 10.1126/sciimmunol.abc3582 PMC7285829 32404436

[33] Junji X, Xiaojing Z, Mingli F, Evan Z, Laurie JM, Zhiqiang Z (2021) DHX15 is required to control RNA virus-induced intestinal inflammation. Cell Rep. 10.1016/j.celrep.2021.109205 10.1016/j.celrep.2021.109205 PMC8276442 34161762

[34] Wang JY, Zhang W, Roehrl VB, Roehrl MW, Roehrl MH (2022) An autoantigen atlas from human lung HFL1 cells offers clues to neurological and diverse autoimmune manifestations of COVID-19. Front Immunol 13:831849. 10.3389/fimmu.2022.831849 35401574 10.3389/fimmu.2022.831849 PMC8987778

[35] Rotondi M, Coperchini F, Ricci G, Denegri M, Croce L, Ngnitejeu ST et al (2020) Detection of SARS-COV-2 receptor ACE-2 mRNA in thyroid cells: a clue for COVID-19-related subacute thyroiditis. J Endocrinol Investig. 10.1007/s40618-020-01436-w 10.1007/s40618-020-01436-w PMC7538193 33025553

[36] Niraj S, Rebecca L, Perminder G (2020) Considerations for target oxygen saturation in COVID-19 patients: Are we under-shooting? BMC Med. 10.1186/s12916-020-01735-2 10.1186/s12916-020-01735-2 PMC7437106 32814566

[37] Kalfaoglu B, Almeida-Santos J, Tye CA, Satou Y, Ono M (2020) T-cell hyperactivation and paralysis in severe COVID-19 infection revealed by single-cell analysis. Front Immunol 11:589380. 10.3389/fimmu.2020.589380 33178221 10.3389/fimmu.2020.589380 PMC7596772

[38] Glinsky G (2020) Harnessing powers of genomics to build molecular maps of coronavirus targets in human cells: a guide for existing drug repurposing and experimental studies identifying candidate therapeutics to mitigate the pandemic COVID-19

[39] Mariana GF, Avantika L, Rita R, Andreas JG, Andrea G, Itziar Martinez G et al (2021) Genome-wide bioinformatic analyses predict key host and viral factors in SARS-CoV-2 pathogenesis. Commun Biol. 10.1038/s42003-021-02095-0 10.1038/s42003-021-02095-0 PMC8128904 34002013

[40] Kartikay P, Suliman Yousef A, Saeed Awad MA, Md Zubbair M, Vijay K (2021) Brain disease network analysis to elucidate the neurological manifestations of COVID-19. Mol Neurobiol. 10.1007/s12035-020-02266-w 10.1007/s12035-020-02266-w PMC7787249 33409839

[41] Beerli C, Yakimovich A, Kilcher S, Reynoso GV, Fläschner G, Müller DJ et al (2019) Vaccinia virus hijacks EGFR signalling to enhance virus spread through rapid and directed infected cell motility. Nat Microbiol 4(2):216–225. 10.1038/s41564-018-0288-2 30420785 10.1038/s41564-018-0288-2 PMC6354922

[42] Kung CP, Meckes DG Jr, Raab-Traub N (2011) Epstein-Barr virus LMP1 activates EGFR, STAT3, and ERK through effects on PKCdelta. J Virol 85(9):4399–4408. 10.1128/jvi.01703-10 21307189 10.1128/JVI.01703-10 PMC3126279

[43] Zhu L, Lee PK, Lee WM, Zhao Y, Yu D, Chen Y (2009) Rhinovirus-induced major airway mucin production involves a novel TLR3-EGFR-dependent pathway. Am J Respir Cell Mol Biol 40(5):610–619. 10.1165/rcmb.2008-0223OC 18978302 10.1165/rcmb.2008-0223OC PMC2677440

[44] Ghasemnejad-Berenji M, Pashapour S (2021) SARS-CoV-2 and the Possible role of Raf/MEK/ERK pathway in viral survival: Is this a potential therapeutic strategy for COVID-19? Pharmacology 106(1–2):119–122. 10.1159/000511280 33011728 10.1159/000511280 PMC7573895

[45] Sciacchitano S, Sacconi A, De Vitis C, Blandino G, Piaggio G, Salvati V et al (2021) H-Ras gene takes part to the host immune response to COVID-19. Cell Death Discov 7(1):158. 10.1038/s41420-021-00541-w 34226505 10.1038/s41420-021-00541-w PMC8256395

[46] Shasha L, Wenli L, Yangzhen C, Liqin W, Wenlin A, Xiaoping A et al (2020) Transcriptome analysis of cepharanthine against a SARS-CoV-2-related coronavirus. Brief Bioinform. 10.1093/bib/bbaa387 10.1093/bib/bbaa387 PMC7929461 33423067

[47] Sarah Musa H, Arabella Musa H, Poorna Manasa B, Habiba Al S, Bassam M, Axel K et al (2021) Systems immunology analysis reveals the contribution of pulmonary and extrapulmonary tissues to the immunopathogenesis of severe COVID-19 patients. Front Immunol. 10.3389/fimmu.2021.595150 10.3389/fimmu.2021.595150 PMC8273737 34262555

[48] Wang JY, Zhang W, Roehrl MW et al (2021) An autoantigen atlas from human lung HFL1 cells offers clues to neurological and diverse autoimmune manifestations of COVID-19. biorxiv. 10.1101/2021.01.24.427965 35401574 10.3389/fimmu.2022.831849 PMC8987778

[49] Mandal M, Mandal S (2021) Bioinformatic approaches for identification of potential repurposable drugs in COVID-19. J Drug Deliv Ther 11(1):13–22

[50] Keunsoo K, Hoo K, Yoonjung C (2020) Tiotropium is predicted to be a promising drug for COVID-19 through transcriptome-based comprehensive molecular pathway analysis. Viruses. 10.3390/v12070776 10.3390/v12070776 PMC7412475 32698440

[51] Mustafa Sait G, Merve A, Emre D, Yusuf Ö, Serdar Ş, Dildar K et al (2021) Rapid and effective vitamin D supplementation may present better clinical outcomes in COVID-19 (SARS-CoV-2) patients by altering serum INOS1, IL1B, IFNg, Cathelicidin-LL37, and ICAM1. Nutrients. 10.3390/nu13114047 10.3390/nu13114047 PMC8618389 34836309

[52] Ceylan H (2021) A bioinformatics approach for identifying potential molecular mechanisms and key genes involved in COVID-19 associated cardiac remodeling. Gene reports 24:101246. 10.1016/j.genrep.2021.101246 34131597 10.1016/j.genrep.2021.101246 PMC8192842

[53] Wen W, Su W, Tang H, Le W, Zhang X, Zheng Y et al (2020) Immune cell profiling of COVID-19 patients in the recovery stageby single-cell sequencing. Cell Discov 6(1):31. 10.1038/s41421-020-0168-9 32377375 10.1038/s41421-020-0168-9 PMC7197635

[54] Stukalov A, Girault V, Grass V, Karayel O, Bergant V, Urban C et al (2021) Multilevel proteomics reveals host perturbations by SARS-CoV-2 and SARS-CoV. Nature 594(7862):246–252. 10.1038/s41586-021-03493-4 33845483 10.1038/s41586-021-03493-4

[55] Jayanta Kumar D, Swarup R, Pietro Hiram G (2021) Analyzing host-viral interactome of SARS-CoV-2 for identifying vulnerable host proteins during COVID-19 pathogenesis. Infect Genet Evol. 10.1016/j.meegid.2021.104921 10.1016/j.meegid.2021.104921 PMC8123524 34004362

[56] Wang Y, Wang X, Luu LDW, Li J, Cui X, Yao H et al (2021) Single-cell transcriptomic atlas of individuals receiving inactivated COVID-19 vaccines reveals distinct immunological responses between vaccine and natural SARS-CoV-2 infection. medRxiv. 10.1101/2021.08.30.2126286334931200

[57] Deepyaman D, Soumita P (2021) Unraveling the molecular crosstalk between Atherosclerosis and COVID-19 comorbidity. Comput Biol Med. 10.1016/j.compbiomed.2021.104459 10.1016/j.compbiomed.2021.104459 PMC8088080 34020127

[58] Zeng C, Hou X, Yan J et al (2021) Leveraging mRNAs sequences to express SARS-CoV-2 antigens in vivo. biorxiv. 10.1101/2020.04.01.01987734981053

[59] Shirvaliloo M (2021) Epigenomics in COVID-19; the link between DNA methylation, histone modifications and SARS-CoV-2 infection. Epigenomics 13(10):745–750. 10.2217/epi-2021-0057 33876664 10.2217/epi-2021-0057 PMC8074570

[60] Guoping L, Xiang H, Lei Z, Qin R, Junyi W, Anying X et al (2020) Assessing ACE2 expression patterns in lung tissues in the pathogenesis of COVID-19. J Autoimmun. 10.1016/j.jaut.2020.102463 10.1016/j.jaut.2020.102463 PMC7152872 32303424

[61] Ellen W, Alexander P, Priscilla LY, Martin S, Qingsong L, Qingwang L et al (2020) Repurposing of kinase inhibitors for treatment of COVID-19. Pharm Res. 10.1007/s11095-020-02851-7 10.1007/s11095-020-02851-7 PMC7417114 32778962

[62] Tsukamoto K, Ohta N, Shirai Y, Emi M (1998) A highly polymorphic CA repeat marker at the human tumor necrosis factor alpha (TNFAα) locus. J Hum Genet 43:278–279. 10.1007/s100380050090 10.1007/s100380050090 9852684

[63] Saleh A, Sultan A, Elashry MA, Farag A, Mortada MI, Ghannam MA et al (2020) Association of TNF-α G-308 a promoter polymorphism with the course and outcome of COVID-19 patients. Immunol Investig. 10.1080/08820139.2020.1851709 10.1080/08820139.2020.1851709 PMC7711738 33228423

[64] Pierzynowska K, Gaffke L, Węgrzyn G (2020) Transcriptomic analyses suggest that mucopolysaccharidosis patients may be less susceptible to COVID-19. FEBS Lett 594(20):3363–3370 32880920 10.1002/1873-3468.13908 PMC7461230

[65] Pouya F, Imani Saber Z, Kerachian MA (2020) Molecular aspects of Co-morbidities in COVID-19 infection. Arch Bone Joint Surg 8(Suppl1):226–230. 10.22038/abjs.2020.47828.2361 32607393 10.22038/abjs.2020.47828.2361 PMC7296607

[66] Chai P, Yu J, Ge S, Jia R, Fan X (2020) Genetic alteration, RNA expression, and DNA methylation profiling of coronavirus disease 2019 (COVID-19) receptor ACE2 in malignancies: a pan-cancer analysis. J Hematol Oncol 13(1):43. 10.1186/s13045-020-00883-5 32366279 10.1186/s13045-020-00883-5 PMC7197362

[67] Choe JW, Jung YK, Yim HJ, Seo GH (2022) Clinical effect of hepatitis B virus on COVID-19 infected patients: a nationwide population-based study using the health insurance review and assessment service database. J Korean Med Sci 37(4):e29. 10.3346/jkms.2022.37.e29 35075828 10.3346/jkms.2022.37.e29 PMC8787805

[68] Lee YR, Kang MK, Song JE, Kim HJ, Kweon YO, Tak WY et al (2020) Clinical outcomes of coronavirus disease 2019 in patients with pre-existing liver diseases: a multicenter study in South Korea. Clin Mol Hepatol 26(4):562–576. 10.3350/cmh.2020.0126 33053932 10.3350/cmh.2020.0126 PMC7641571

[69] Sarin SK, Choudhury A, Lau GK, Zheng MH, Ji D, Abd-Elsalam S et al (2020) Pre-existing liver disease is associated with poor outcome in patients with SARS CoV2 infection; the APCOLIS study (APASL COVID-19 liver injury spectrum study). Hep Intl 14(5):690–700. 10.1007/s12072-020-10072-8 10.1007/s12072-020-10072-8 PMC7334898 32623632

[70] Samad A, Jafar T, Rafi JH (2020) Identification of angiotensin-converting enzyme 2 (ACE2) protein as the potential biomarker in SARS-CoV-2 infection-related lung cancer using computational analyses. Genomics 112(6):4912–4923. 10.1016/j.ygeno.2020.09.002 32916258 10.1016/j.ygeno.2020.09.002 PMC7831469

[71] Hossain MG, Akter S, Uddin MJ (2022) Expression profile of SARS-COV-2 entry receptor ACE2 in the hepatocellular carcinoma and its impact on Covid-19 patients. J Microbiol Biotechnol Food Sci 11(4):e5140. 10.55251/jmbfs.5140

[72] Mohammed A, Paranji N, Chen PH, Niu B (2021) COVID-19 in Chronic liver disease and liver transplantation: a clinical review. J Clin Gastroenterol 55(3):187–194. 10.1097/mcg.0000000000001481 33394628 10.1097/MCG.0000000000001481 PMC7959867

[73] Xiang TD, Zheng X (2021) Interaction between hepatitis B virus and SARS-CoV-2 infections. World J Gastroenterol 27(9):782–793. 10.3748/wjg.v27.i9.782 33727770 10.3748/wjg.v27.i9.782 PMC7941862

[74] Chen X, Jiang Q, Ma Z, Ling J, Hu W, Cao Q et al (2020) Clinical characteristics of hospitalized patients with SARS-CoV-2 and hepatitis B virus Co-infection. Virol Sin 35(6):842–845. 10.1007/s12250-020-00276-5 32839868 10.1007/s12250-020-00276-5 PMC7444863

[75] Fajgenbaum DC, June CH (2020) Cytokine storm. N Engl J Med 383(23):2255–2273. 10.1056/NEJMra2026131 33264547 10.1056/NEJMra2026131 PMC7727315

[76] Wang H, Ma S (2008) The cytokine storm and factors determining the sequence and severity of organ dysfunction in multiple organ dysfunction syndrome. Am J Emerg Med 26(6):711–715. 10.1016/j.ajem.2007.10.031 18606328 10.1016/j.ajem.2007.10.031

[77] Sahu KK, Cerny J (2021) A review on how to do hematology consults during COVID-19 pandemic. Blood Rev 47:100777. 10.1016/j.blre.2020.100777 33199084 10.1016/j.blre.2020.100777 PMC7648889

[78] McConnell MJ, Kondo R, Kawaguchi N, Iwakiri Y (2022) Covid-19 and liver injury: role of inflammatory endotheliopathy, platelet dysfunction, and thrombosis. Hepatol Commun 6(2):255–269. 10.1002/hep4.1843 34658172 10.1002/hep4.1843 PMC8652692

[79] Dawood DRM, Salum GM, El-Meguid MA (2022) The impact of COVID-19 on liver injury. Am J Med Sci 363(2):94–103. 10.1016/j.amjms.2021.11.001 34752738 10.1016/j.amjms.2021.11.001 PMC8571104

[80] Wang H, Sun Q, Zhao W, Qi L, Gu Y, Li P et al (2015) Individual-level analysis of differential expression of genes and pathways for personalized medicine. Bioinformatics (Oxford, England) 31(1):62–68. 10.1093/bioinformatics/btu522 25165092 10.1093/bioinformatics/btu522

[81] Burska AN, Roget K, Blits M, Soto Gomez L, van de Loo F, Hazelwood LD et al (2014) Gene expression analysis in RA: towards personalized medicine. Pharmacogn J 14(2):93–106. 10.1038/tpj.2013.48 10.1038/tpj.2013.48 PMC3992869 24589910

