# Supplementary figures and images for: A systems biology approach for investigating significantly expressed genes among COVID-19, hepatocellular carcinoma, and chronic hepatitis B
Source: Egypt J Med Hum Genet. 2022 Oct 20;23(1):146. doi: 10.1186/s43042-022-00360-3 (PMC9584277; doi:10.1186/s43042-022-00360-3)

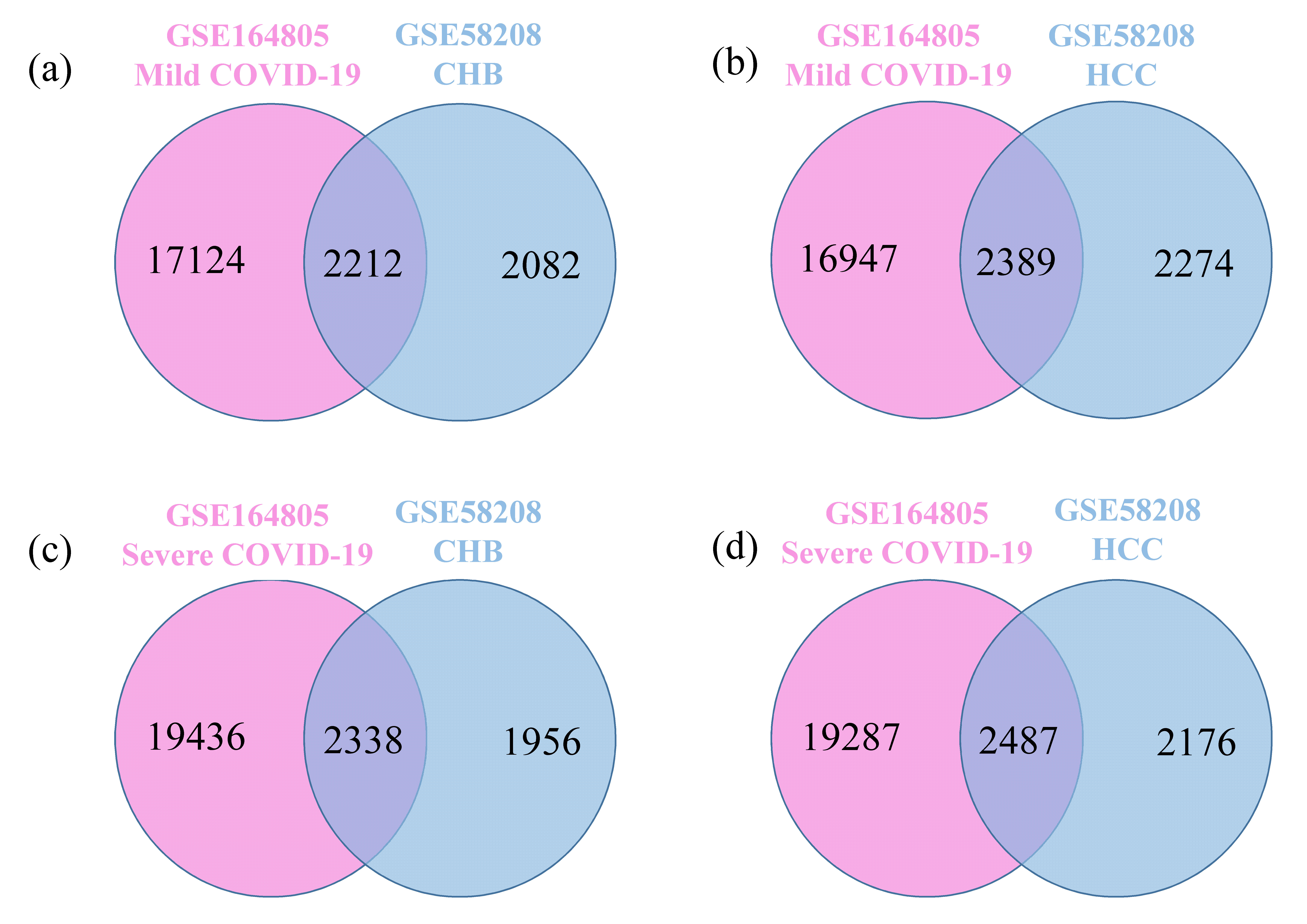

Supplement: Supplementary file 1 — Additional file 1. Fig 1: Venn diagrams depicting the common DEGs between mild COVID-19 vs. CHB, mild COVID-19 vs. HCC, severe COVID-19 vs. CHB, severe COVID-19 vs. HCC datasets [file 43042_2022_360_MOESM1_ESM.jpg]

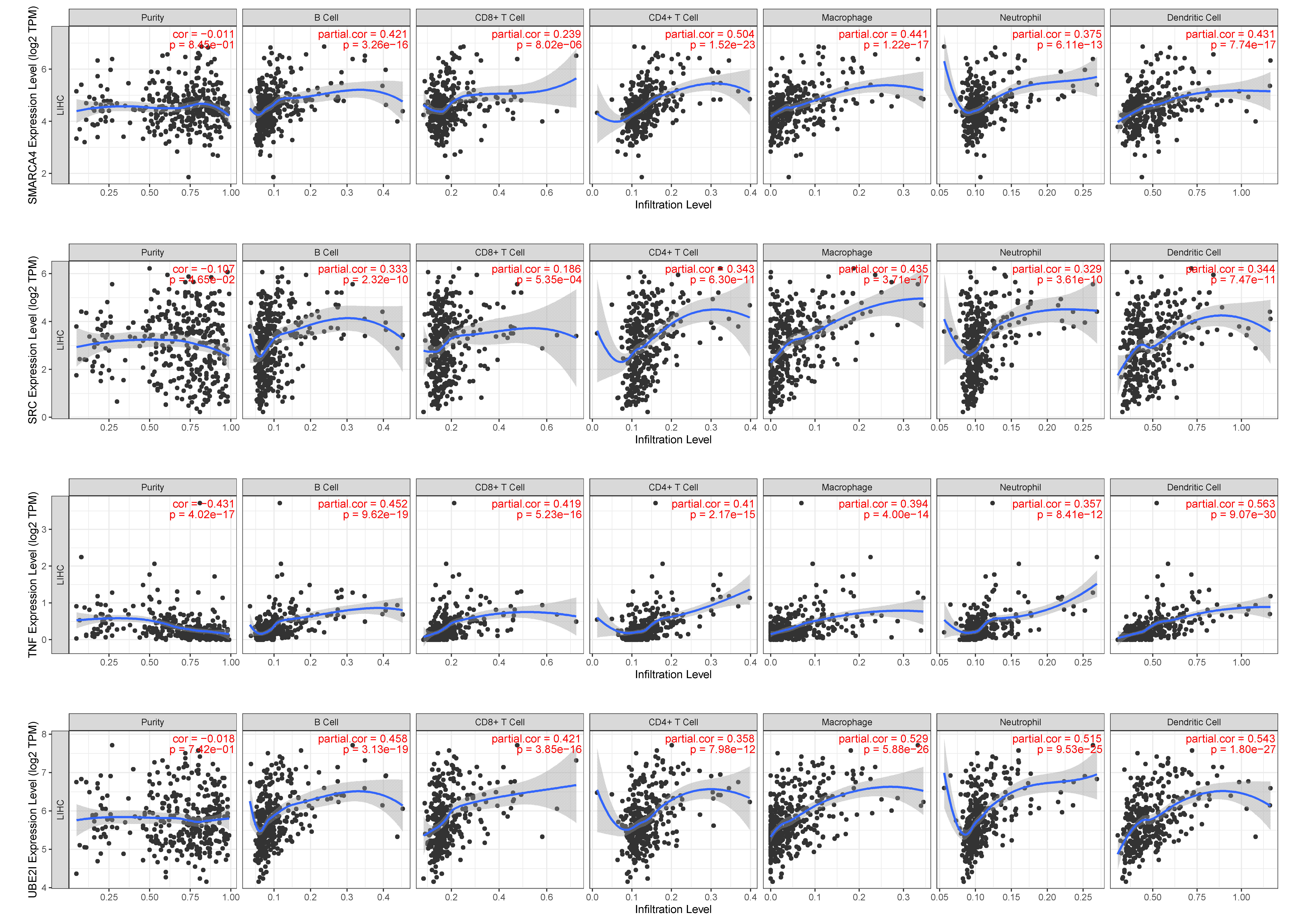

Supplement: Supplementary file 2 — Additional file 2. Fig 2: Correlation between the identified DEGs and immune cell infiltration (TIMER); p value<0.05 represented statistically significant [file 43042_2022_360_MOESM2_ESM.zip › SuppFigure 2-1R2.jpg]

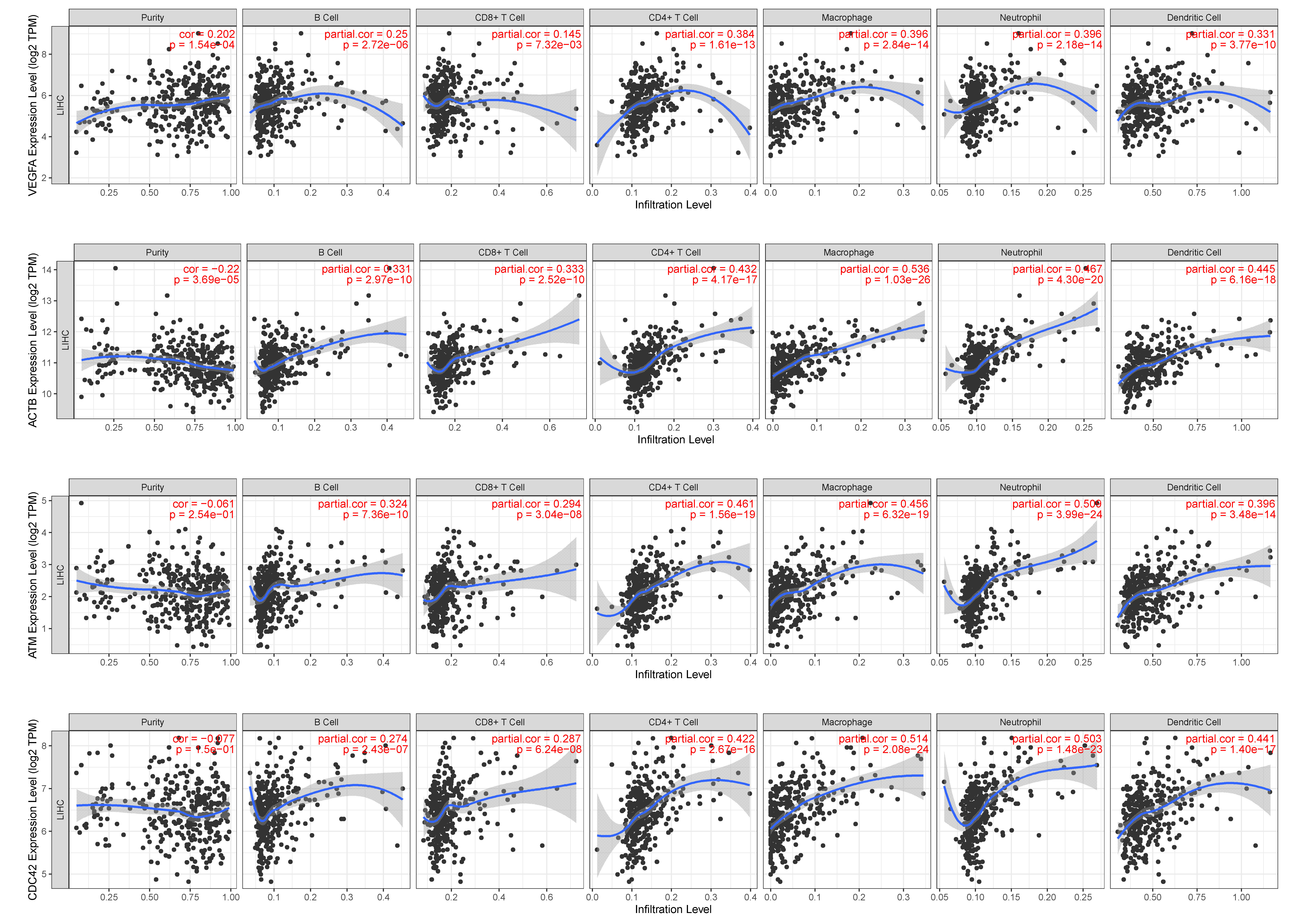

Supplement: Supplementary file 2 — Additional file 2. Fig 2: Correlation between the identified DEGs and immune cell infiltration (TIMER); p value<0.05 represented statistically significant [file 43042_2022_360_MOESM2_ESM.zip › SuppFigure 2-2R2.jpg]

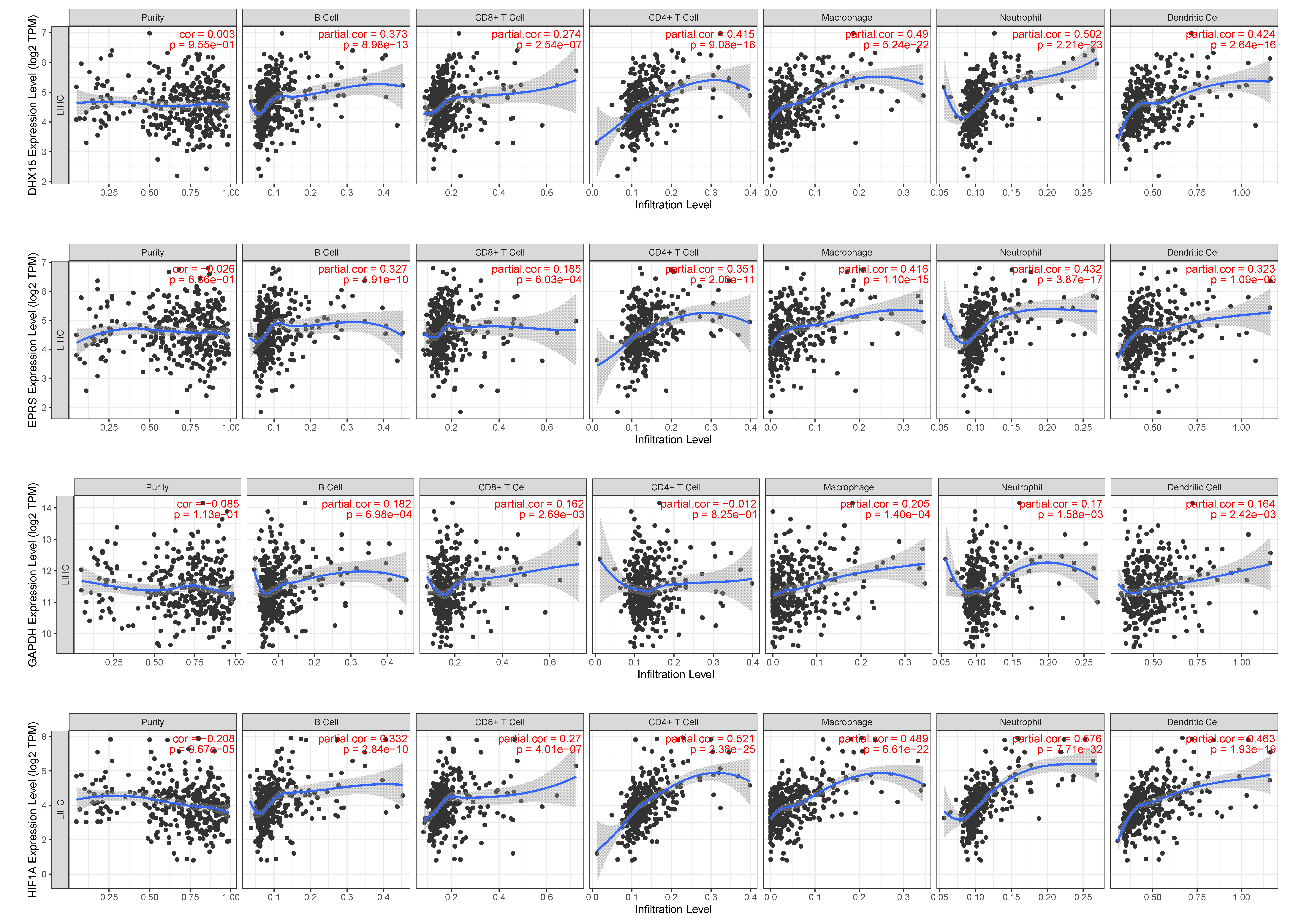

Supplement: Supplementary file 2 — Additional file 2. Fig 2: Correlation between the identified DEGs and immune cell infiltration (TIMER); p value<0.05 represented statistically significant [file 43042_2022_360_MOESM2_ESM.zip › SuppFigure 2-3R2.jpg]

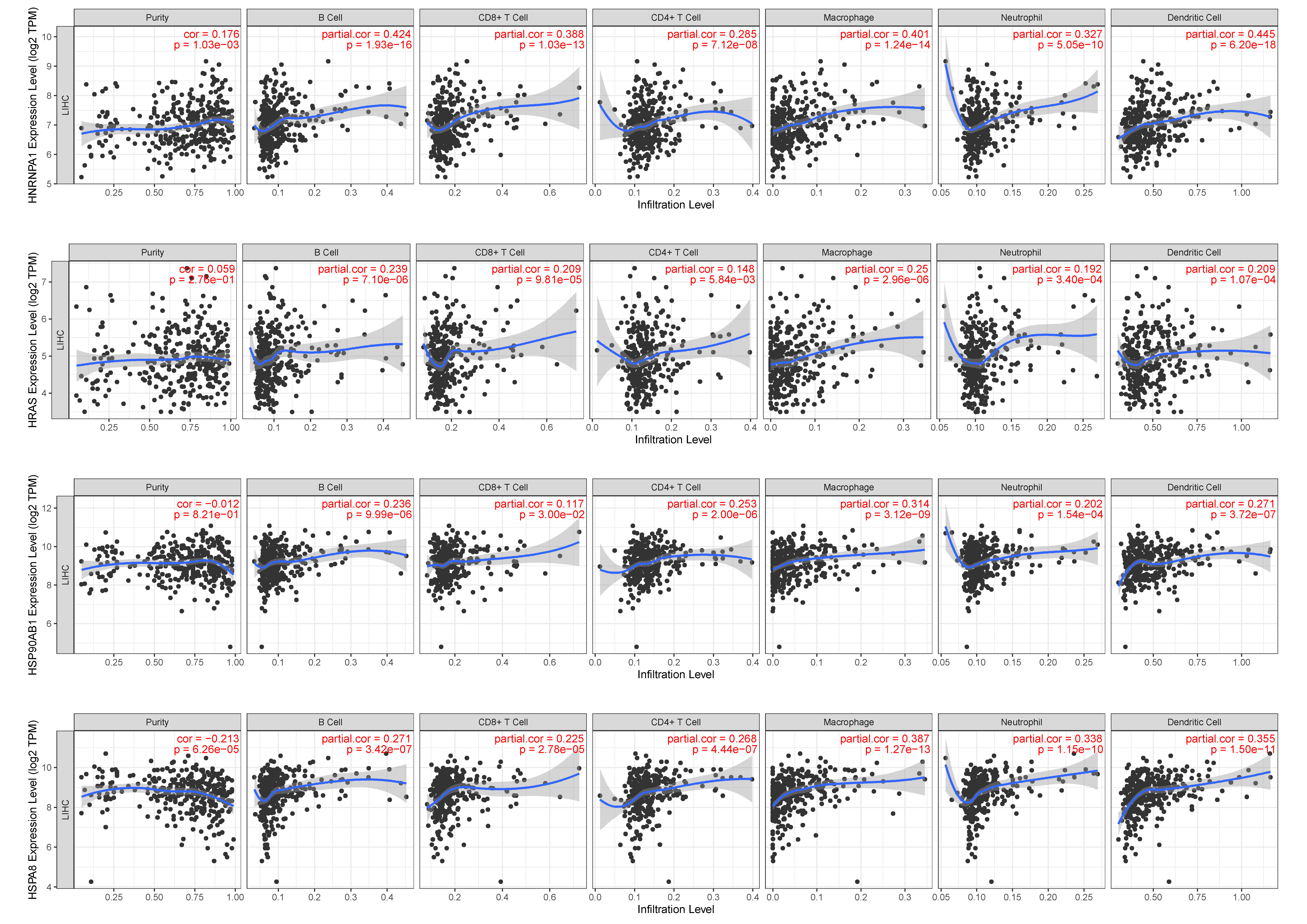

Supplement: Supplementary file 2 — Additional file 2. Fig 2: Correlation between the identified DEGs and immune cell infiltration (TIMER); p value<0.05 represented statistically significant [file 43042_2022_360_MOESM2_ESM.zip › SuppFigure 2-4R2.jpg]

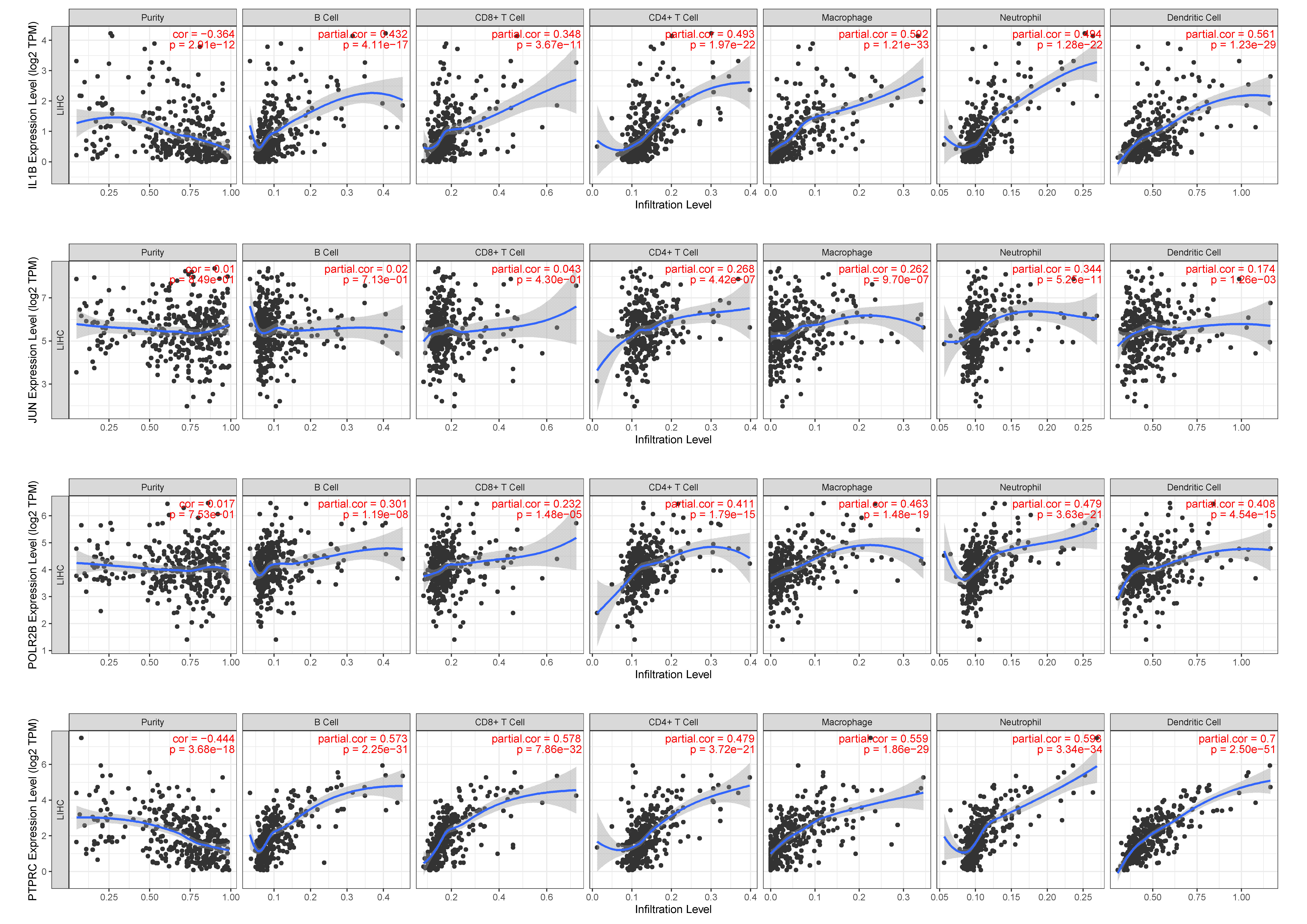

Supplement: Supplementary file 2 — Additional file 2. Fig 2: Correlation between the identified DEGs and immune cell infiltration (TIMER); p value<0.05 represented statistically significant [file 43042_2022_360_MOESM2_ESM.zip › SuppFigure 2-5R2.jpg]

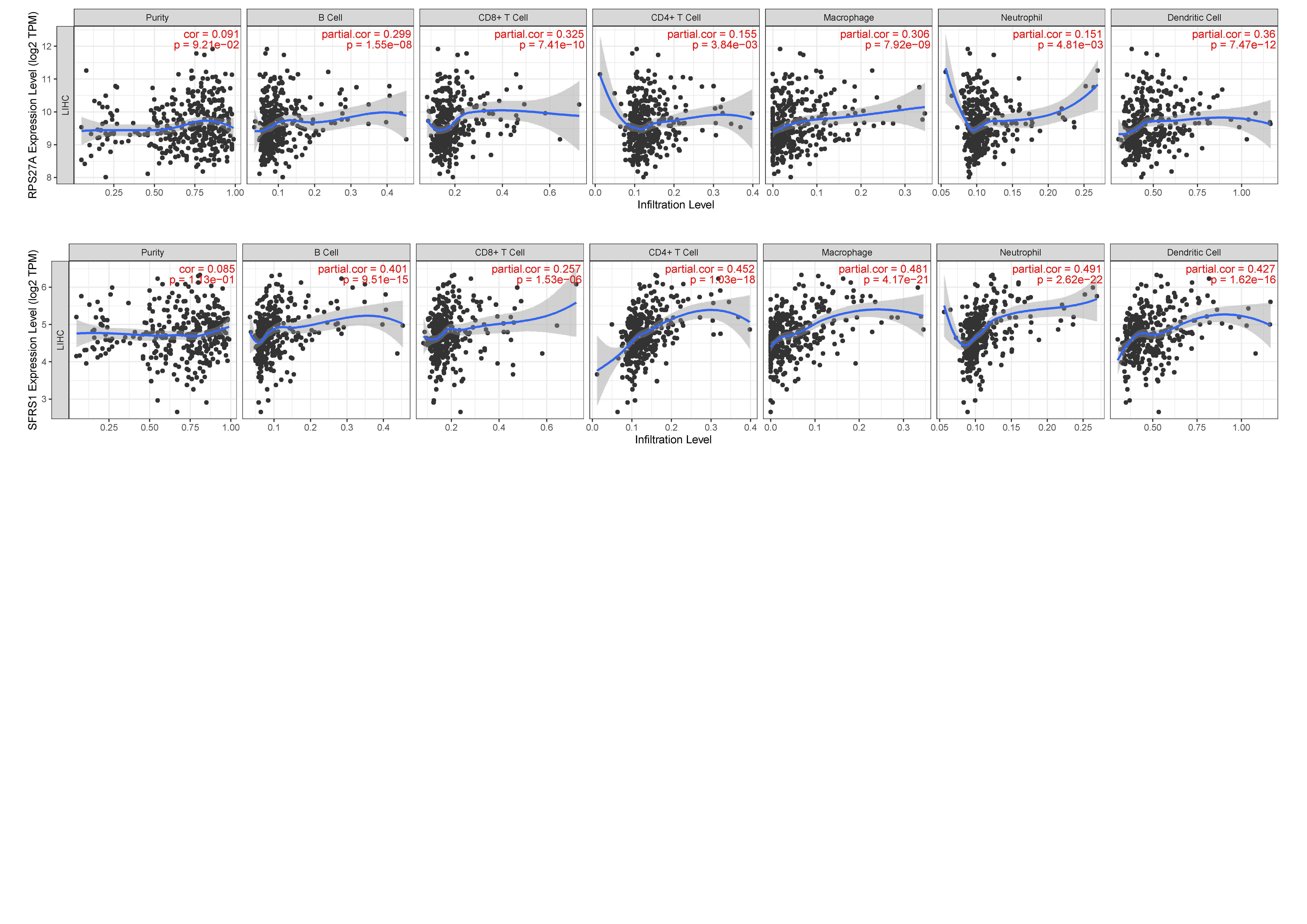

Supplement: Supplementary file 2 — Additional file 2. Fig 2: Correlation between the identified DEGs and immune cell infiltration (TIMER); p value<0.05 represented statistically significant [file 43042_2022_360_MOESM2_ESM.zip › SuppFigure 2-6R2.jpg]
